# Reinforced Concrete Plates under Impact Load—Damage Quantification

**DOI:** 10.3390/ma13204554

**Published:** 2020-10-14

**Authors:** Marcus Hering, Franz Bracklow, Silke Scheerer, Manfred Curbach

**Affiliations:** Faculty of Civil Engineering, Institute of Concrete Structures, D-01062 Dresden, Germany; franz.bracklow@tu-dresden.de (F.B.); Silke.Scheerer@tu-dresden.de (S.S.); Manfred.Curbach@tu-dresden.de (M.C.)

**Keywords:** impact, reinforced concrete, strengthening, carbon concrete

## Abstract

A large number of impact experiments have been carried out at the Technische Universität Dresden in recent years in several research projects. The focus was on reinforced concrete plates on the one hand and on subsequently strengthened reinforced concrete plates on the other hand. Based on these investigations, two fundamental tasks arose: (1) finding an objective description of the damage of components made of steel reinforced concrete that had previously been subjected to an impact load and (2) quantification of the effect of a subsequently applied strengthening layer. In this paper we will focus on both. At first, the experimental conditions and program as well as the used drop tower facility at the Otto Mohr Laboratory of the Technische Universität Dresden are briefly explained. In the summary presentation of the main test results, the focus is on the observed component damage. Based on the observations, an approach for a damage description is presented. To define global damage, the stiffness of the investigated structural components before and after the impact event is used. At the end of the paper, the potential of the method, but also gaps in knowledge and research needs are discussed.

## 1. Introduction

For the investigation of reinforced concrete (RC) structures under impact load in laboratory tests, displacements, deformations and support forces, accelerations or general descriptions of the observed damage are often used [1,2,3,4,5,6,7,8,9,10,11]. The measured data are analyzed, evaluated, compared and often subsequently used for further investigations, e.g., for numerical simulations of the experiment [12,13]. A difficult aspect considering RC structures subject to impact is the evaluation of the damage resulting from the loading scenario. In order to be able to estimate this damage, the previously mentioned measured values are used, but a quantitative statement is difficult in many cases.

**Figure 1:**
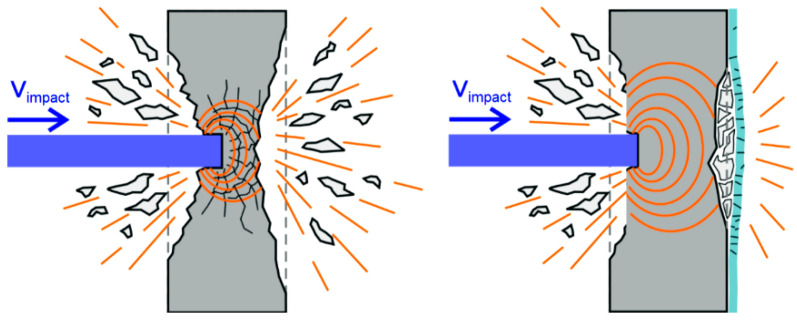
Behavior of an unprotected RC component (**left**) and of a strengthened concrete element (**right**) under impact; original graphic: by M. Butler (see Curosu et al. [14]), modified by M. Hering.

In a research project, conducted in the frame of the Research Training Group GRK 2250 “Mineral-bonded composites for enhanced structural impact safety”, a further aspect has now been added to the description of damage of impacted RC structures. Within this project A5 “Strengthening of plane RC elements against impact on the impact-far side” the principle of a subsequent strengthening of RC structures by thin reinforcement layers to increase their resistance against impact loading was investigated, [14]. For strengthening, the focus was on mineral-bonded composites. Such strengthening layers consist of two components: on the one hand the matrix material and on the other hand the reinforcement material. Short fibers as well as continuous fiber fabrics or meshes made of steel wires can be used as reinforcing material. The reference plates were tested in the frame of the project “Structural behaviour under impact loading by the impacting container (aircraft tanks)”, e.g., [10,11,15], founded by BMWi. A basic variant study with different strengthening materials was carried out in the AiF project “Reinforced concrete structures strengthened with Textile Reinforced Concrete (TRC) for impact loading” [16]. Within the GRK the structural behavior overall and in detail were investigated with more depth and with focus on the basic mechanisms and damage processes under consideration of the interplay of different material combinations. In general, it should also be investigated which combinations of mineral bonded matrix and reinforcement material are most efficient to strengthen a structure against an impact load. In this publication, only those strengthening layers are considered which were applied to the side of the component facing away from the impact, see Figure 1.

A quantification of a combined local and global component damage was previously carried out, among others, in Just et al. [10] in verbally defined damage classes or based on the deformations of the damaged components, Jonas et al. [4]. In order to quantify the performance of the applied strengthening layer, a continuous description of the damage was first formulated and examined on the basis of experiments with RC plates. Above all, a method for determining the input variables had to be developed. This method was then evaluated by experiments with reference RC plates and subsequently strengthened RC plates. This allowed a quantitative evaluation of the used strengthening layers.

Some aspects of the experimental results presented in this article have already been published in Hering et al. [15] and—depth consideration—in Hering [17]. These publications also contain detailed descriptions of the experimental setup, the specimens used, the experimental procedure and the evaluation of the experiments also can be found. For the sake of completeness, the basic facts are briefly outlined in Section 2.

## 2. Specimens, Experimental Setup and Summary of the Experiments

### 2.1. Specimen

The basic test specimens were reinforced concrete (RC) plates with outer dimensions of 1.5 m × 1.5 m × 0.2 m. These plates were made of normal strength concrete C35/45. As reinforcement, BSt500(B) steel with a diameter of 8 mm was used. The reinforcement was arranged crosswise parallel to the long sides of the plate with a distance of 100 mm between the bars. The upper and lower reinforcement layers were identical. The concrete cover was 25 mm, see Hering et al. [15] and Hering [17].

**Figure 2:**
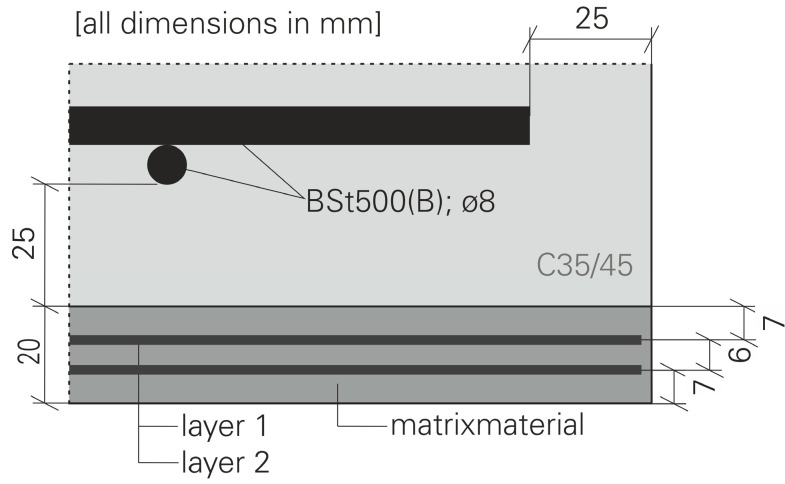
Basic structure of the strengthening layer; Hering [17].

The focus of the investigations was on increasing the impact resistance through the use of subsequently applied strengthening layers out of mineral bonded cement composites. For this reason, five RC reference plates and several similar RC plates for strengthening were produced. Before the strengthening was carried out, the side of the plate to be strengthened was pre-treated by roughening with exposed aggregate concrete paper and then washing out the cement. Different strengthening layers were considered in the course of the investigations. Within the scope of this publication, the strengthening with carbon reinforced concrete according to [18,19] shall be exemplarily dealt with. The strengthening was realized with a 20 mm thick layer of fine grain concrete (Pagel TF10, [18]). Two layers of TUDALIT-BZT1-TUDATEX [18] (BZT1) were embedded in this layer. The basic structure of the strengthening layer is shown in Figure 2.

### 2.2. Experimental Schedule

**Figure 3:**
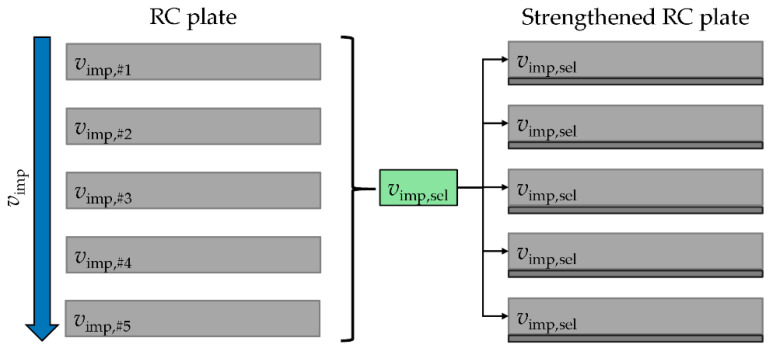
Schematic experimental schedule; Hering [17].

The experimental investigations were separated into two parts. On the one hand, the damage behavior of RC plates was considered. For this purpose, the already described RC plates were examined with different impact velocities. The impact velocities were increased step by step. With the lowest impact velocity, the specimen was only slightly damaged. This damage, which was determined purely subjectively, was increased by raising the impact velocity until the plate was perforated by the impactor, see Figure 3 and [15].

From the five impact velocities considered, an impact speed *v*_imp,sel_ was selected at which a significant damage of the RC plate had occurred. With this impact velocity the subsequently strengthened RC plates were tested. With this approach, on the one hand it was possible to examine how the damage of the test specimen changes as a result of the increasing impactor velocity. On the other hand, it was also possible to classify the damage in the strengthened RC plates compared to the five unstrengthened reference RC plates. Thus, it is possible to evaluate the strengthening effect.

In the context of this publication, only one possible strengthening variant will be discussed to illustrate the basic procedure. The main focus of this article is the description of the damage that has occurred.

### 2.3. Experimental Setup

**Figure 4:**
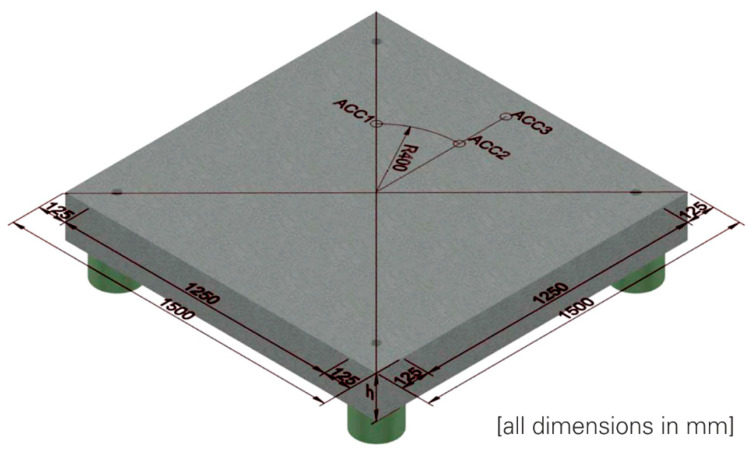
Schematic illustration of the support conditions and positioning of the acceleration sensors; Hering [17].

The impact loading of the plates was carried out in the accelerated configuration of the drop tower facility of the Otto Mohr Laboratory at the Technische Universität Dresden [10,11]. The support conditions of the plates during the experiment are shown schematically in Figure 4. A four-point support was used [15,17].

The impact load was applied by a steel impactor with a diameter of 100 mm, a length of 380 mm and a weight of 21.66 kg. The impactor nose was flat [15,17]. The impactor was accelerated by compressed air. The charging pressure was used as control variable.

Table 1 shows the test and impact velocities of the impactor in the case of the reference specimens and the strengthened plate.

## 3. Preliminary Considerations on the Description of Global Damage

**Figure 5:**
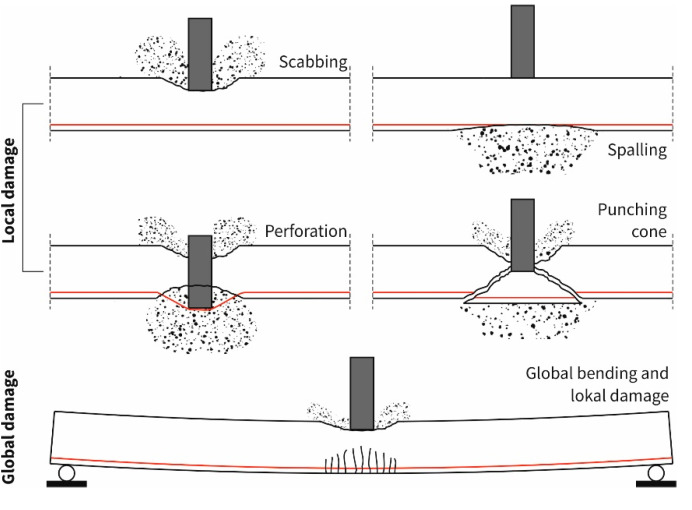
Schematic illustration of the different types of damage; graphic: S. Scheerer.

The damage can be differentiated in general into a local and a global damage. In the case of local damage, scabbing, spalling, punching shear failure and perforation can be distinguished. These types of damage are shown in Figure 5 based on [20]. A quantification of this damage is possible by the penetration depth of the impactor into the component, the damage radius, the damaged area or the mass of the broken out material. In the context of this publication we will concentrate on the mass of the material broken out of the specimen by scabbing and spalling to describe the amount of local damage. “Global” means the load-bearing behavior including damage mechanisms of the overall structure or the entire component. Here, the support conditions or the presence of adjacent components can have a relevant influence (an example is shown in the bottom of Figure 5).

To quantify the global damage, only qualitative degrees of damage have been used so far, see e.g., Just et al. [10]. The use of such degrees of damage makes it possible to classify the respective component damage. However, comparative observations are only possible to a limited extent, because the classification into damage grades has so far only been subjectively. Therefore, it is extremely difficult, if not impossible, to assess the strengthening effect or the effectiveness of subsequently applied strengthening layers, because the evaluation always lies in the eye of the one evaluating, and subjective influences cannot be excluded.

Based on this problem, a separate quantitative description of the damage was developed on the basis of the structural stiffness of the plate [17]. For this purpose, the stiffness of the structure before the experiment (*K*_1_) and the stiffness after the experiment (*K*_2_) is used, see Equations (1) and (2).

*Φ*_global_ = *ΔK*/*K*_1_ (1)

*ΔK* = *K*_1_−*K*_2_ (2)

with: *Φ*_global_ degree of damage, related to the global structural behavior.

One problem that now had to be solved, was the measurement of structural stiffness of the plate. Ideally, this stiffness is determined by means of static experiments. However, the experimental investigation of an already tested plate in the drop tower is not recommended, since the initial state of the test specimen should be undamaged. For this reason, the detour via the measurement of the natural frequency (*ω*_E_) was used to draw conclusions about the component’s stiffness. This in turn can be calculated via the eigenfrequency (*f*_E_), see Equation (3). If the component mass involved in the oscillations (*m*_eff_) is known, the structural stiffness of the plate can be calculated according to Equation (4).

The component mass *m*_eff_ depends on its geometry and the support conditions. However, the influence of the support conditions can be neglected if the static system does not change due to the impact experiment, see Hering [17].

f_E_ = 2·π·ω_E_ (3)

*K* = *m*_eff_·(*ω*_E_)^2^ (4)

**Figure 6:**
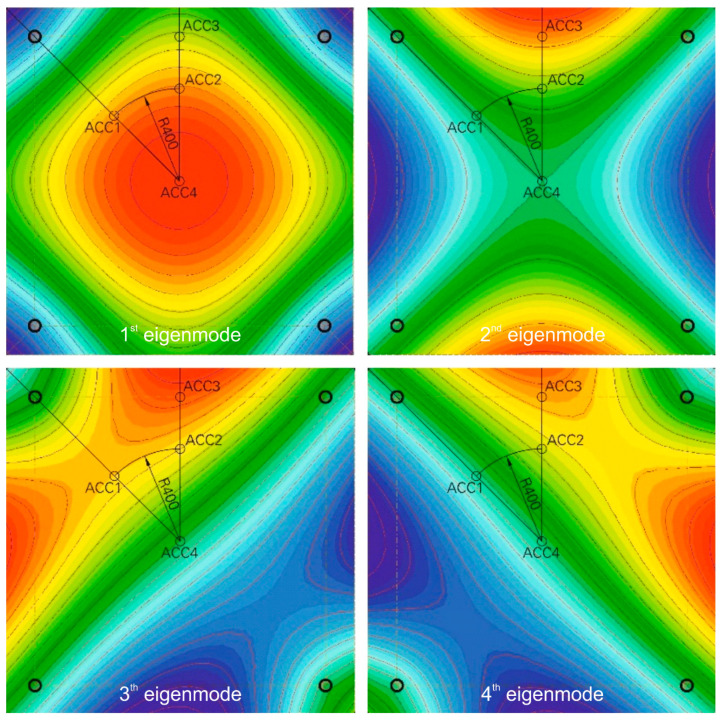
Eigenmodes of the reference RC plate with a four-point support in the corners according to Figure 4; graphic: Hering [17].

Acceleration sensors (ACC1 to ACC4) were used to determine the eigenfrequency of the four-point supported plates. The positions of these sensors are shown in Figure 4 and also in Figure 6, but there without ACC4, because it was placed in the middle on the bottom of the plate. In preparation for the experimental investigations, finite element method (FEM) simulations were carried out with the FEM software SOFiSTiK to determine the position of the sensors. Thereby the eigenmodes of the plate were evaluated. In Figure 6, the first four eigenmodes are shown.

By selecting the sensor positions as shown in Figure 4 and Figure 6, it was possible to detect the first eigenmode of the plate, which was used to determine the plate’s stiffness before and after the impact experiment, see Table 2.

## 4. Experimental Results—Occurring Damage

### 4.1. Local Damage—Scabbing and Spalling

**Figure 7:**
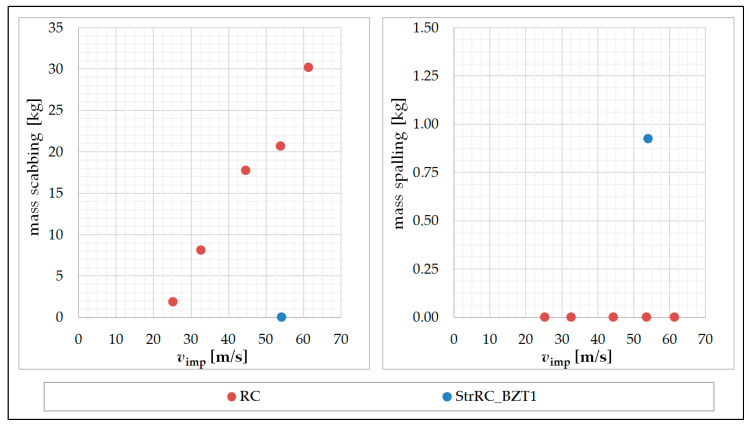
Scabbing (**left**) and spalling masses (**right**) of the reference plates RC_1–5 and the strengthened plate StrRC_BZT1 after impact; graphic: M. Hering.

As described before, the approach of the evaluation of the local damage is based on mass reduction of the plate due to scabbing and spalling. The corresponding fragment masses, weighted after the experiment, are summarized in Table 3 and visualized in Figure 7. In the left diagram can be seen, that the mass of material that broke out of the plate as a result of scabbing increased significantly as the impact velocity of the impactor increased. Based on the right diagram it can be noted that no spalling occurred in the reference RC plates.

In comparison to the non-strengthened RC plates, it was found that local damage due to scabbing on the rear side of the RC plate (StrRC_BZT1) was completely prevented by the subsequent applied strengthening layer. However, it was also shown that this experiment revealed a small amount of material due to spalling on the front side of the plate.

### 4.2. Local Damage—Punching Shear Failure

**Figure 8:**
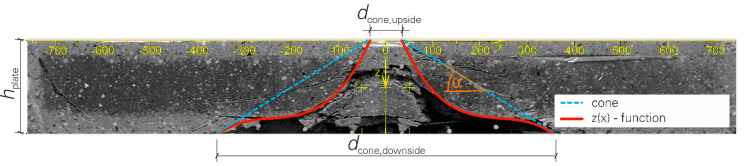
Fracture body due to punching shear failure; real cone and cubic function in comparison; graphic: Hering [17].

After the plates were subjected to impact loading in the drop tower facility, the specimens were cut in the middle. This procedure made it possible to get a view of the damage inside the plates. The shape of the fracture body inside the plate is usually described as a cone [21,22,23], but in our experiments, the shape deviated significantly from a cone, see Figure 8. It was measured by means of digital image analysis. The resulting data showed that the shape of the fracture body can be approximated very well by a third-degree polynomial function. As already described in [21,22], the fracture body showed a very high symmetry. In Figure 8, both the cone and the polynomial function z(*x*), which can be represented by Equation (5), are displayed.

z(x) = *p*_3_·*x*^3^ + *p*_2_·*x*^2^ + *p*_1_·*x*^1^ + *p*_0_ (5)

**Figure 9:**
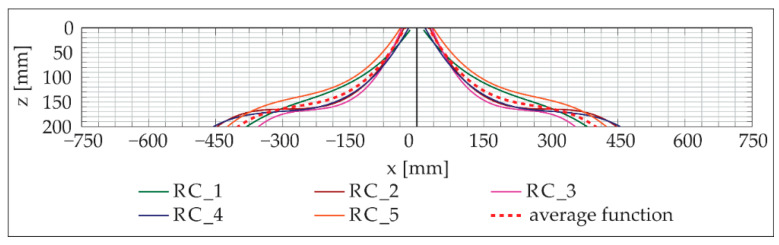
Comparison of the fracture bodies depending on the different impactor velocities; graphic: M. Hering [17].

In Figure 9, the determined functions of the fracture bodies of the individual RC plates are shown. Furthermore, the mean value of these functions was determined and also displayed. When looking at Figure 9, it can be seen that in the considered velocity range (Table 3), no influence of the impactor’s velocity on the shape of the fracture body can be determined. Its shape shows some scattering, but no systematics can be derived for the specimens under consideration.

**Figure 10:**
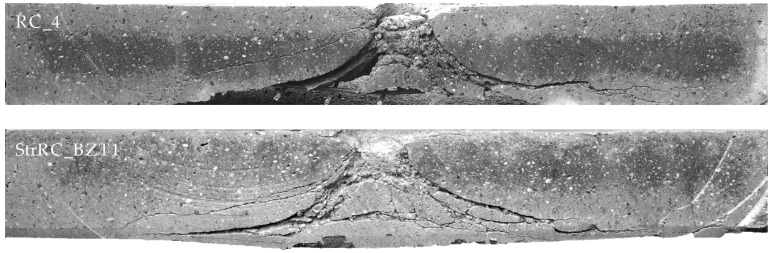
Comparison of the saw cuts of the plates RC_4 and StrRC_BZT1; photos: M. Hering.

In Figure 10, the saw cuts of the RC plate RC_4 and the strengthened plate StrRC_BZT1 were compared. For both specimens the velocity of the impactor *v*_imp,sel_ was about 54 m/s. The fracture bodies are clearly visible. The main difference between the two specimens is that in the plate StrRC_BZT1 the subsequently applied strengthening layer retains the material crushed by the impact inside the specimen. This prevents material from being removed from the back of the plate (scabbing). However, the basic type of failure—punching failure—was not changed by the applied strengthening layer, but the intensity of the damage that occurred could be significantly reduced.

### 4.3. Global Damage

To specify the global damage according to Equation (1), the eigenfrequency of the specimens was determined at six separate points in time during experiment execution, see Table 4, using a half-round impactor head to excite the plate. For this purpose, this impactor head was struck by hand on the upper side of the plate. This process was recorded by the applied acceleration sensors.

**Figure 11:**
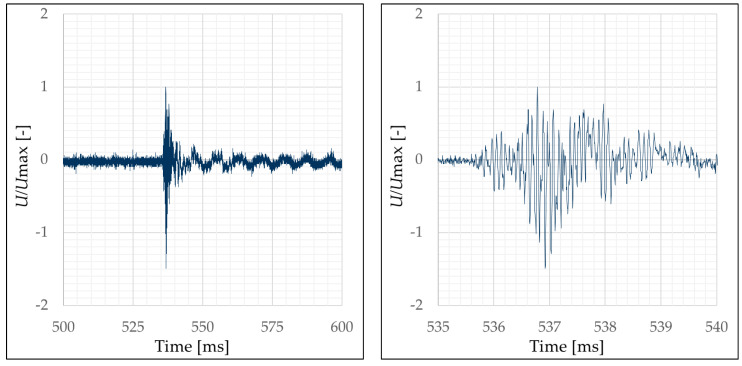
Signal measured with acceleration sensor ACC1, uncalibrated but normalized with the maximum value (detailed visualization of the maximum and minimum peak values in the right diagram), complete signal (**left**) visualization of the first peak (**right**); graphic: Hering [17].

**Figure 12:**
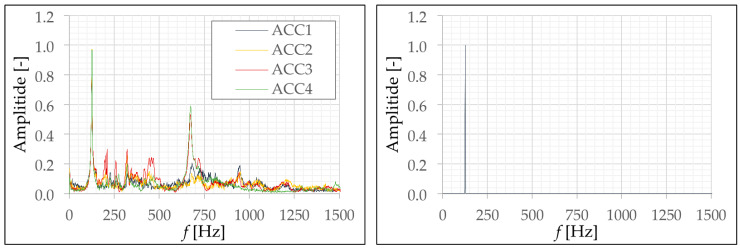
Amplitude-frequency diagram resulting from FFT (**left**) and amplitude-frequency diagram modified with Equation (6) (**right**); graphic: M. Hering [17].

An example of such a measurement is shown in Figure 11. However, it is not yet possible to determine the first eigenfrequency from this diagram. Therefore, the signal has to be processed by a Fast Fourier Transformation (FFT). The FFT delivers the measured amplitudes of the signal to the corresponding frequencies. Such an amplitude-frequency diagram is shown as an example in Figure 12 (left diagram). There, the first, i.e., the lowest eigenfrequency can be easily recognized by the maximum amplitude peak. Higher eigenfrequencies are also visible. These can be recognized by the peaks in the higher frequency range.

For the systematic evaluation it is important to be able to determine the first eigenfrequency clearly. For this reason, the Equation (6) was used for signal modification and amplification. By multiplying the amplitude-frequency curve with itself, the amplitudes are amplified. This means that existing peaks are significantly increased and areas with low amplitudes are significantly reduced. The multiplication of the FFT of the individual sensor signals with each other leads to the fact that only the signal is maintained, which was measured by all acceleration sensors. According to Table 2 this is the first eigenfrequency. The modified amplitude-frequency diagram of the FFT of the accelerometer signals is shown as an example in Figure 12 (right diagram). Here, the first natural frequency can be clearly identified. Thus, a systematic evaluation is now possible.

FFT(ACC1, …, ACC4) = FFT(ACC1)^2^·FFT(ACC2)^2^·FFT(ACC3)^2^·FFT(ACC4)^2^ (6)

The eigenfrequencies, which were determined with the different stimulations, are summarized in Table 5. In order to use *Φ*_global_ according to Equation (1) the mean value was calculated from the values *Φ*_global,St1,St6_, *Φ*_global,St2,St5_ and *Φ*_global,St3,St4_. The damage of the test specimens was determined under the same support conditions before and after the experiment. The determined values *Φ*_global_ of the test samples were summarized in Table 5.

**Figure 13:**
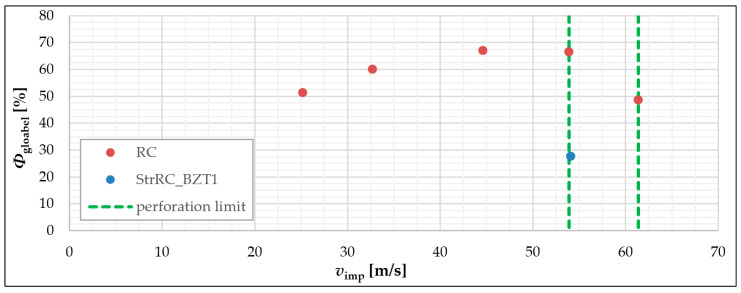
Representation of the global damage (*Φ*_global_) in relation to the impactor velocity (*v*_imp_); graphic: Hering [17].

In Figure 13 the determined degrees of damage *Φ*_global_ are plotted over the velocity of the impactor during the corresponding experiment. Here it can be seen how the degree of damage increases with increasing impactor speed. This trend continues until the impactor velocity nears the perforation velocity. This can be assumed to be between 53.9 m/s and 61.4 m/s for the examined RC plates, see [15,17]. In the range of this perforation velocity, the degree of damage decreases. A significant decrease in the degree of damage can be observed for the plate RC_5 with the highest impactor velocity *v_imp_* = 61.4 m/s. The comparison of RC_4 and StrRC_BZT1 shows that a significant reduction of the damage degree was achieved by applying a strengthening layer on the side of the plate facing away from the impact.

## 5. Conclusions and Outlook

The presented work introduces a methodology to quantify systematically the damage of RC plates without and with subsequent applied strengthening layer caused by an impact load. For this purpose, known approaches and parameters described in this article like the mass of break-out due to scabbing and spalling as well as the global damage index and the eigenfrequency were taken into account. It could be shown, that the description of damage using the degree of damage *Φ*_global_ is basically feasible, and with the help of the described damage characteristics, a systematic consideration of the effect of an impact load on RC plates is possible. But there are some important remarks and also knowledge gaps which have to be closed in the future:

(1) At first it should be stated that an explicit classification of damage degree depending on the impactor impact velocity is only possible to a limited extent. This can be easily shown on the example of the RC_1 and RC_5 test specimens. For both plates, the global degrees of damage *Φ*_global_ are about 50%. But despite the same numerical values for the damage degree, the boundary conditions of these experiments differ considerably with regard to the velocity of the impactor and the associated local damage of the component. Thus, it has to be concluded that *Φ*_global_ cannot be used as an exclusive criterion for the damage description. Instead, the combination of global and local damage of the structure must always be considered in context. The interaction of both types of damage was underestimated.
(2) It should be noted that the investigations carried out are of selective nature. Scattering in the experimental results due to varying material parameters or geometries were not specifically recorded and can therefore not be evaluated in general.
(3) It has to be examined whether an extension of the damage description has to be made with regard to the possible change of the support conditions due to the impact. In the experiments presented, the laboratory environment ensured that the static system before and after the impact event was identical. In reality, a change of the support conditions due to an extreme loading event has to be considered.
(4) It is also not yet possible to estimate the susceptibility to errors of the described method, as the number of experiments does not allow this.
(5) An objective description and evaluation of the effect of subsequently applied strengthening layers is possible using the different damage characteristics. The method is particularly suitable for direct comparison of the effectiveness of different material combinations which can be used for strengthening.

In sum, it can be stated that the presented methodology to predict the structural damage resulting from an impact event is currently still in its initial stage. It could be shown that it works well in principle. However, the robustness of the method could not be estimated due to the small number of conducted experiments. The approach presented is a first step towards the description of damage. However, intensive research work still needs to be invested in its further development.

## Figures and Tables

**Table 1:** Specimen identification, impact velocities and energies.

Plate Label	Plate Number According to [15,17]	Impactor Velocity * v_imp_ [m/s]	Impactor Energy * [J]
RC_1	PL124	25.2	6877.5
RC_2	PL121	32.7	11,580.4
RC_3	PL120	44.6	21,542.6
RC_4	PL122	53.9	31,463.4
RC_5	PL123	61.4	40,828.7
StrRC_BZT1	PL125	54.1	31,697.4

* at the time of impact.

**Table 2:** Accelerometers and corresponding measured eigenmodes.

Eigenmode	ACC1	ACC2	ACC3	ACC4
**Eigenmode 1**	X	X	X	X
**Eigenmode 2**	O	X	X	O
**Eigenmode 3**	X	X	X	O
**Eigenmode 4**	O	X	X	O

X = eigenmode is measured, O = eigenmode is not measured.

**Table 3:** Masses of the plates before and after the experiment and masses of scabbing/spalling failure; M. Hering [17].

Plate Label	Impactor Velocity ** v_imp_ [m/s]	Plate Mass before Impact *m*_1_ [kg]	Plate Mass after Impact *m*_2_ [kg]	Scabbing Mass *m*_scabb_ [kg]	Spalling Mass *m*_spall_ [kg]
RC_1	25.2	988.00	986.10	1.90	0.00
RC_2	32.7	990.00	981.90	8.10	0.00
RC_3	44.6	996.00	978.26	17.74	0.00
RC_4	**53.9** **	1002.00	981.32	20.68	0.00
RC_5	61.4	958.22	928.00	30.20	0.00
StrRC_BZT1	54.1	1092.00	1091.08	0.00	0.92

** v_RC_4_ = *v*_imp,sel_.

**Table 4:** Time points of the eigenfrequency determination with designation.

Stimulation No.	Position of the Plate
St1	free hanging before the impact experiment
St2	free lying before the impact experiment
St3	clamped on load cells before the impact experiment
St4	clamped on load cells after the impact experiment
St5	free lying after the impact experiment
St6	free hanging after the impact experiment

**Table 5:** Measured eigenfrequencies for each stimulation *f_E,Stx_* and global damage *Φ_global_*; Hering [17].

Specimen Label	*f*_E,St1_ [Hz]	*f*_E,St2_ [Hz]	*f*_E,St3_ [Hz]	*f*_E,St4_ [Hz]	*f*_E,St5_ [Hz]	*f*_E,St6_ [Hz]	*Φ*_global_ [%]
RC_1	311.0	65.0	110.5	80.5	49.8	182.5	51.3
RC_2	308.5	##,#	115.5	82.0	##,#	169.0	60.1
RC_3	311.0	116.8	126.0	81.5	73.3	138.0	67.0
RC_4	317.0	99.3	120.0	76.0	60.5	159.5	66.5
RC_5	307.5	91.0	117.0	85.5	79.0	169.5	48.6
StrRC_BZT1	391.0	64.4	91.8	89.0	60.3	232.5	27.7

##,# ambiguous data.
